# Supplementary material for: Association between Survival and Time of On-Scene Resuscitation in Refractory Out-of-Hospital Cardiac Arrest: A Cross-Sectional Retrospective Study
Source: Int J Environ Res Public Health. 2021 Jan 9;18(2):496. doi: 10.3390/ijerph18020496 (PMC7826551; doi:10.3390/ijerph18020496)
Supplement: Supplementary file 1 [file ijerph-18-00496-s001.pdf]

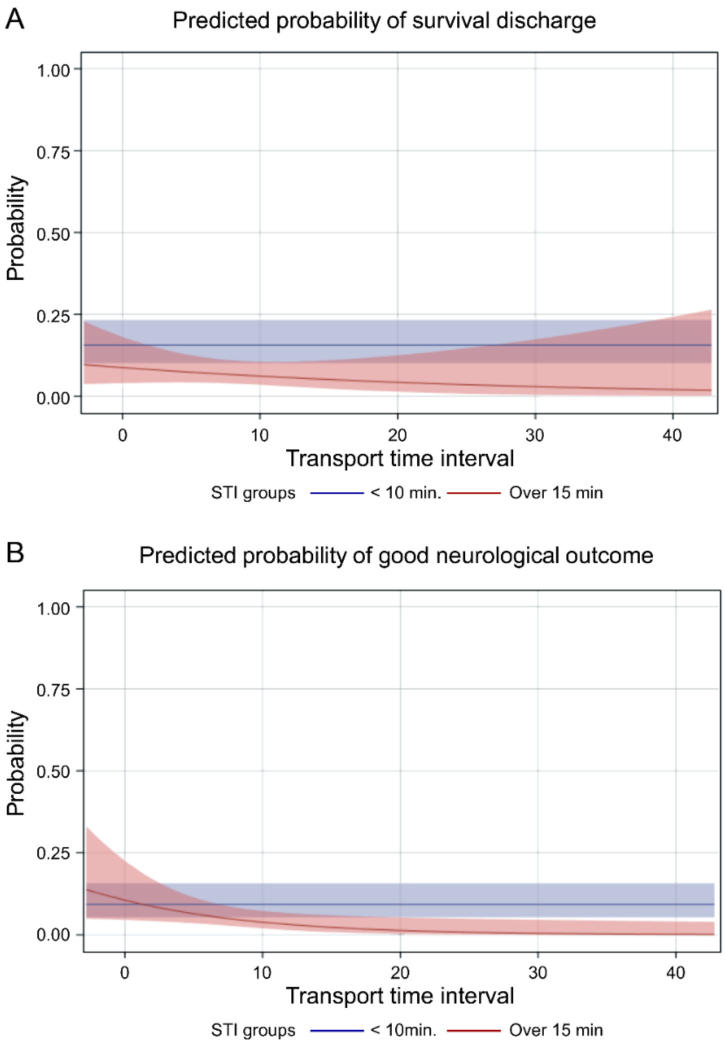

Figure S1. Interaction effects between transport time intervals and scene time interval (STI) groups on survival discharge and good neurological outcome with 95% confidence intervals.

Table S1. Multivariable logistic regression analysis for outcomes in without prehospital ROSC patients.

Variables	Survival discharge				Good neurological outcome			
	OR	95% CI	aOR	95% CI	OR	95% CI	aOR	95% CI
STI ≥ 15 min (< 15 min)	0.52	(0.23-1.18)	0.45	(0.18-1.16)	0.80	(0.31-2.11)	0.38	(0.22-0.67)
Age, years	0.96	(0.93-0.99)	0.95	(0.92-0.98)	0.95	(0.92-0.98)	0.95	(0.94-0.97)
Male	1.17	(0.38-3.59)	1.13	(0.32-3.98)	1.05	(0.29-3.81)	0.60	(0.30-1.22)
Bystander CPR done	0.48	(0.21-1.10)	0.62	(0.26-1.49)	0.75	(0.28-1.96)	1.23	(0.71-2.15)
Public location (non–public)	0.97	(0.43-2.20)	0.78	(0.31-1.97)	1.46	(0.56-3.84)	1.36	(0.78-2.38)
RTI, minute	0.92	(0.80-1.06)	0.89	(0.75-1.05)	0.97	(0.83-1.13)	0.88	(0.79-0.98)

TTI, minute	1.02	(0.98-1.07)	1.03	(0.98-1.08)	1.04	(0.99-1.09)	1.04	(1.01-1.07)
Supraglottic airway (bag valve mask)	0.51	(0.19-1.35)	0.55	(0.19-1.59)	0.50	(0.16-1.58)	0.91	(0.48-1.75)
Endotracheal intubation (bag valve mask)	2.97	(0.85-10.39)	3.57	(0.9-14.17)	2.51	(0.6-10.55)	2.81	(1.10-7.21)

Abbreviations: ROSC, return on spontaneous circulation; OR, Odds ratio; CI, confidence interval; aOR, adjusted odds ratio; STI, scene time interval; RTI, response time interval; TTI, transport time interval; CPR, cardiopulmonary resuscitation..

All references are shown in parentheses.
